# Population expansion and gene flow in *Giardia duodenalis* as revealed by triosephosphate isomerase gene

**DOI:** 10.1186/s13071-015-1084-y

**Published:** 2015-09-15

**Authors:** Seow Huey Choy, Mohammed A. K. Mahdy, Hesham M. Al-Mekhlafi, Van Lun Low, Johari Surin

**Affiliations:** Department of Parasitology, Faculty of Medicine, University of Malaya, 50603 Kuala Lumpur, Malaysia; Department of Parasitology, Faculty of Medicine and Health Science, Sana’a University, Sana’a, Yemen; Tropical Disease Research Center, University of Science and Technology, Sana’a, Yemen; Institute of Biological Sciences, Faculty of Science, University of Malaya, 50603 Kuala Lumpur, Malaysia

**Keywords:** Genetic diversity, Population genetic study, Malaysia, Global population

## Abstract

**Background:**

*Giardia duodenalis* is a protozoan parasite that can cause significant diarrhoeal diseases. Knowledge of population genetics is a prerequisite for ascertaining the invasion patterns of this parasite. In order to infer evolutionary patterns that could not be uncovered based on the morphological features, a population genetic study with the incorporation of molecular marker was carried out to access the genetic structure of *G. duodenalis* isolated from the Malaysian population and the global populations.

**Methods:**

A total of 154 samples positive for *Giardia*, collected from different Malaysian communities, were subjected to DNA amplification and sequencing targeting three genetic loci (*tpi, gdh,* and *bg*). The *tpi* sequences together with sequences from the global data obtained from the NCBI GenBank were used for genetic diversity analyses including identification of haplotypes, haplotype diversity, nucleotide diversity, Tajima’s D and Fu and Li’s D, gene flow and genetic differentiation tests.

**Results:**

Analysis of the Malaysian and global data showed that assemblages A, B, and E (the most prevalent assemblages in humans and animals), have different levels of genetic diversity. Assemblage B had the highest level of both haplotype diversity and nucleotide diversity, followed by assemblage E. The analysis also revealed population expansion and high gene flow in all assemblages. No clear genetic structure was observed across five continents (i.e., the Americas, Europe, Asia, Australia and Africa). However, median joining network of assemblage B formed a cluster that was exclusively isolated from Asia while other haplotypes were well dispersed across the continents.

**Conclusions:**

This study provides new insight into the genetic diversity of *Giardia* assemblages in different geographical regions. The significant result shown by gene flow and genetic differentiation analyses as well as test of neutrality among the populations should have brought a clearer picture to the dynamics and distribution of *Giardia* infection.

## Background

*Giardia* is a gastrointestinal flagellate with a worldwide distribution affecting both developed and developing countries. It is also well known as a ubiquitous parasite of a wide range of vertebrates from mammals to birds and amphibians. *Giardia* trophozoites produce energy by bacterial anaerobic pathways, lack of subcellular organelles such as mitochondria, peroxisomes and have only rudimentary Golgi apparatus [1, 2]. The exhibition of these prokaryotic features had led many to believe that *Giardia* represents the transitional form between prokaryotes and eukaryotes which developed eukaryotic features before mitochondrial symbiosis [3]. Alongside with phylogenetic analysis of ribosomal RNA, *Giardia* had been described in literature as ‘early branching eukaryotes’ and was commonly used as a model system to study the development of basic cellular processes and evolution of eukaryotes [2]. However, over the past decade, the advancement in phylogenetic studies has shown that the placement of *Giardia* near the base of ribosomal RNA tree was attributed to long-branch attraction caused by rapid evolution of the organisms [4, 5]. Emerging evidences from genomic and ultrastructural studies indicate that all extant groups of amitochondrial organisms have genetic marker of mitochondria and mitochondria-derived organelles such as mitosome in *Giardia.* Thus it is suggested that, the microbial organisms are probably more advanced eukaryotes that underwent loss or modification of complex structures as a consequence of their adaptation to parasitic life style [4, 6].

Despite its ubiquitous presence, the genus of *Giardia* which used to have over 50 described species [7] now comprises six highly host specific members with only *G. duodenalis* being isolated from humans and other mammalian hosts. It contributes to approximately 5 % of gastroenteritis incidences in developed countries and as high as 15–55 % in developing countries [8]. This species can be further classified genetically into at least seven assemblages (A-H) with assemblages A and B known to be associated with human and many other animal infections including domestic animals and wildlife while others are host restricted assemblages infecting dogs (C and D), cats (F), hoofed livestock (E), rodents (G) and marine animals (H) [9].

DNA-based genotyping approaches have been widely applied in epidemiology and epizootiology of parasites. From the perspective of population ecology, molecular approaches provide a means for any parasitologist to understand processes such as transmission of parasites, patterns of speciation, evolution of host specificity and spread of drug resistance [10]. The evolutionary potential of parasite is driven by the distribution, flow and degree of genetic diversity along with the magnitude of diversity recombination among genomes [11]. The aim of the present study was to assess the genetic variation of *G. duodenalis* isolated from Malaysia as well as to understand the transcontinental distribution of the parasite genotypes by comparing with sequences recovered from other countries based on *triosephosphate isomerase* (*tpi*) gene.

## Methods

A total of 154 *G. duodenalis* isolates collected from different places in Malaysia were subjected to DNA extraction and amplification with subsequent sequencing targeting the *tpi*, *glutamate dehydrogenase* (*gdh*), and *beta-giardin* (*bg*) genes as indicated previously [12]. The Malaysian sequences of the three loci were used for the assessment of genetic diversity of *G. duodenalis* in Malaysia. Ethical approval: The protocol that involved the sample collection was approved by the Medical Ethics Committee of the University of Malaya Medical Centre (Ref. no: 788.74 and 878.19). Informed consent was obtained from the participants or their parents/guardians before the sample collection was done.

For the population genetic analysis for the worldwide dataset, *tpi* gene was chosen as the candidate as it produced the highest number of amplifications compared to the other two genes and had the relatively longer length of nucleotide sites for sufficient analysis. In addition, molecular characterization analysis using *tpi* was well established [13] and the sequences were readily available in NCBI GenBank. Thus, searches were carried out to retrieve as many *tpi* sequences as possible from NCBI GenBank database. Of the 1465 *tpi* sequences available on NCBI GenBank, only sequences with known sources were selected. A total of 932 sequences (119 sequences from Malaysia and 813 sequences from other countries) were enrolled in the analyses. The sequences were trimmed and sorted into assemblages by visual inspection according to the positions of the single nucleotide polymorphisms (SNPs) using multiple alignments implemented by Clustal W [14] in BioEdit Sequence Alignment Editor Programme [15]. In order to obtain maximum length and maximum number of sequences with matching and continuous coverage for analyses, sequences that were too short and not in the common cover region were omitted. Besides, sequences with heterogeneous nucleotide were removed from the analyses.

A Median Joining Network analysis were performed with all individual *tpi* sequences obtained from previous study [12] and GenBank database to generate haplotype networks between closely related sequences using the program SplitsTree [16] according to assemblages (i.e. A, B and E). Due to small number of sequences available for assemblage C, D, F, G and H, analyses for these isolates were not performed. The sequences were further classified into five populations according to continents (North and South America, Europe, Asia, Australia and Africa) to understand the distribution of *G. duodenalis* genotypes across different regions. Sequences of Malaysia generated from *tpi* gene comprised isolates of assemblages A and B obtained from different states in Malaysia (including both West and East Malaysia) were analysed within its own subset to access the genetic variation and the pattern of genotypes distribution.

Genetic diversity analysis including identification of haplotypes, haplotype diversity (Hd), nucleotide diversity (Pi), Tajima’s *D* (D) and Fu and Li’s *D* (D*), gene flow and genetic differentiation tests were performed using DnaSP v.5.10.01 [17]. The level of genetic differentiation indicated by fixation index (FST) with values ranging from 0 to 1 was rated as FST > 0.25 (huge differentiation), 0.15 to 0.25 (great differentiation), 0.05 to 0.15 (moderate differentiation) and FST < 0.05 (negligible differentiation) [18]. Huge differentiation would imply great separation between two populations while negligible differentiation would imply little divergence and free spreading between two populations. The point of reference for the level of gene flow was referred as high gene flow (Nm > 1), intermediate gene flow (0.25–0.99) and low gene flow (<0.25) [19].

## Results

### Genetic diversity and haplotype networks

Genetic diversity assessment of Malaysian isolates using different genes i.e. *tpi*, *gdh* and *bg* showed that all the three markers produced consistent results, with sequences of assemblage B having the higher level of both haplotype diversity (Hd) and nucleotide diversity (Pi) compared to assemblage A (Table 1). Comparison of nucleotide diversity of the different genes revealed highest level of diversity in *tpi* gene followed by *gdh* and *bg* genes. However, in regards to the haplotype diversity, *bg* gene had the highest diversity followed by *tpi* and *gdh* genes.

**Table 1:** Genetic diversity of *G. duodenalis* of Malaysian population among three loci

Locus	Data set	No of sequences	No of sites	S	h	Hd	Pi
*gdh*	Assemblage A	19	408	2	2	0.10500	0.00052
	Assemblage B	34	408	8	15	0.90600	0.00599
*bg*	Assemblage A	44	486	8	6	0.58400	0.00235
	Assemblage B	23	486	10	11	0.92900	0.00433
*tpi*	Assemblage A	72	476	10	8	0.23500	0.00098
	Assemblage B	47	489	30	27	0.90700	0.00830

Number of polymorphic sites (S), number of haplotypes (h), haplotype diversity (Hd), nucleotide diversity (Pi)

**Fig. 1:**
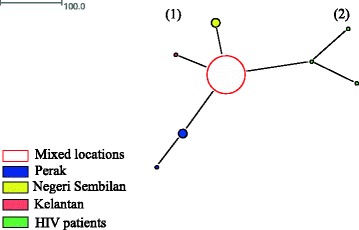
Median joining network of assemblage Asequences from Malaysian population

**Fig. 2:**
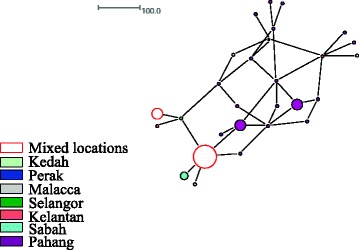
Median joining network of assemblage B sequences from Malaysian population

The 119 *tpi* sequences from Malaysia in which assemblage A comprised eight haplotypes among 72 isolates and assemblage B comprised 27 haplotypes among 47 isolates) were included in the Median Joining Network analysis. Haplotype network of assemblage A formed two distinct networks. The first and the largest network comprised all the haplotypes except three haplotypes that were included in the second network and were isolated from HIV patients (Fig. 1). Inspections of the SNPs and phylogenetic analysis (data not shown) revealed that these three haplotypes belonged to sub-assemblage AI while the rest of the haplotypes in the first network belonged to sub-assemblage AII. However, for assemblage B, no distinct networks were formed and clustering according to the sub-structuring of assemblage (e.g. sub-assemblage BIII and BIV) were not seen (Fig. 2). Both assemblages A and B did not show clear geographical clustering in the distribution of the haplotypes.

**Fig. 3:**
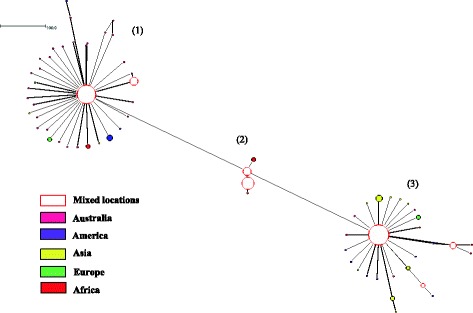
Median joining network of assemblage A sequences of worldwide population

**Fig. 4:**
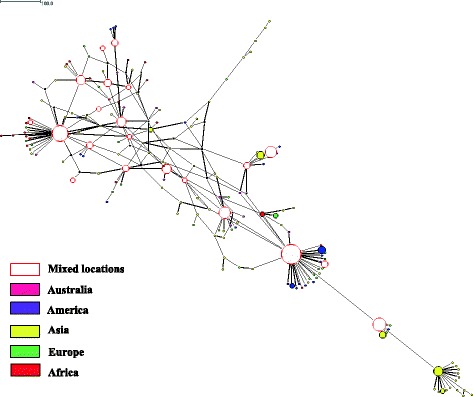
Median joining network of assemblage B sequences of worldwide population

**Fig. 5:**
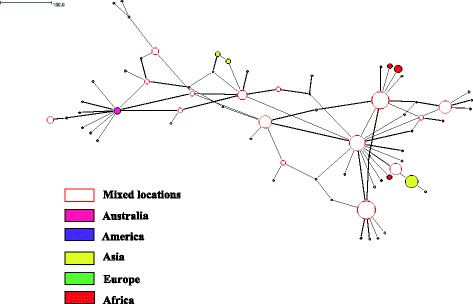
Median joining network of assemblage E sequences of worldwide population

With respect to the worldwide data set, a total of 67, 155 and 66 haplotypes were inferred from sequences of assemblages A, B and E respectively. In terms of genetic diversity, assemblage B had the highest level of both haplotype diversity and nucleotide diversity, followed by assemblage E and assemblage A (Table 2). All of the assemblages across the continents had large haplotype diversity, with an overall diversity ranging from Hd = 0.78 to Hd = 0.97. The European population displayed highest nucleotide diversity for assemblage A (0.01279) and assemblage E (0.1119) whereas the Asian population exhibited the highest nucleotide diversity for assemblage B (0.01216). Three distinct networks from 284 sequences of assemblage A were demonstrated in the median joining network analysis (Fig. 3). Distinct SNPs pattern could be seen in the sequences of these networks. Nevertheless, phylogenetic analysis did not clearly show clustering correspond with the networks and with the sub-structuring of assemblage A (i.e. Sub-assemblages AI, AII and AIII). Instead, haplotypes of sub-assemblage AI and AIII shared the same network 1 (data not shown). Separation according the continents was not seen in the networks of assemblage A. However, it was shown that median joining network of assemblage B for 324 sequences formed a network that was exclusively isolated from Asia (yellow) while other haplotypes were well dispersed across the continents (Fig. 4). For median joining network analysis of the 205 sequences of assemblage E, no distinct network and sub-structuring with the assemblage were observed (Fig. 5). The haplotype with the largest circle in the haplotype network of Malaysian population were predicted to be the ancestral haplotype of assemblages A (Fig. 1) and B (Fig. 2) in Malaysia. However, the ancestral haplotype cannot be identified in the global populations as there were many high and low frequency haplotypes disseminated throughout the network.

**Table 2:** Genetic diversity and test of neutrality of *G. duodenalis* for worldwide population

Population	No of sequences	No of sites	S	h	Hd	Pi	D	D*
Assemblage A								
Australia	36	476	51	31	0.98254	0.00704	−2.63963*	−4.81625*
America	74	476	21	14	0.58312	0.00314	−1.98395*	−4.24360*
Asia	104	476	18	14	0.46004	0.00218	−2.04851*	−3.40876*
Europe	33	476	30	10	0.80682	0.01279	−0.63810	−0.60189
Africa	37	476	23	14	0.84234	0.00599	−1.65001	−1.89922
Overall	284	476	100	67	0.78072	0.00564	−2.51918*	−7.40589*
Assemblage B								
Australia	28	489	25	26	0.99471	0.00882	−1.18578	−2.34957
America	65	489	51	33	0.93365	0.00931	−1.92659*	−4.16927*
Asia	131	489	86	86	0.97510	0.01216	−2.01663*	−5.90951*
Europe	60	489	48	39	0.91073	0.00867	−1.97667*	−4.54163*
Africa	40	489	42	30	0.96795	0.00858	−2.06524*	−4.28121*
Overall	324	489	155	176	0.96600	0.01083	−2.40897*	−8.98197*
Assemblage E								
Australia	32	468	34	29	0.99194	0.01059	−1.55162	−1.98732
America	21	468	11	11	0.81905	0.00435	−1.16746	−1.24885
Asia	79	468	30	30	0.91853	0.00539	−1.82865*	−3.99122*
Europe	11	468	17	11	1.00000	0.01119	−0.43806	−0.62712
Africa	62	468	42	10	0.84234	0.00563	−2.36766*	−5.72396*
Overall	205	468	85	66	0.82443	0.00709	−2.39843*	−8.00707*

Number of polymorphic sites (S), number of haplotypes (h), haplotype diversity (Hd), nucleotide diversity (Pi), Tajima’s *D* (D), Fu and Li’s *D* (D*) tests. Value with asterisks (*) indicate statically significant result (p-value < 0.05)

### Test for neutrality

**Fig. 6:**
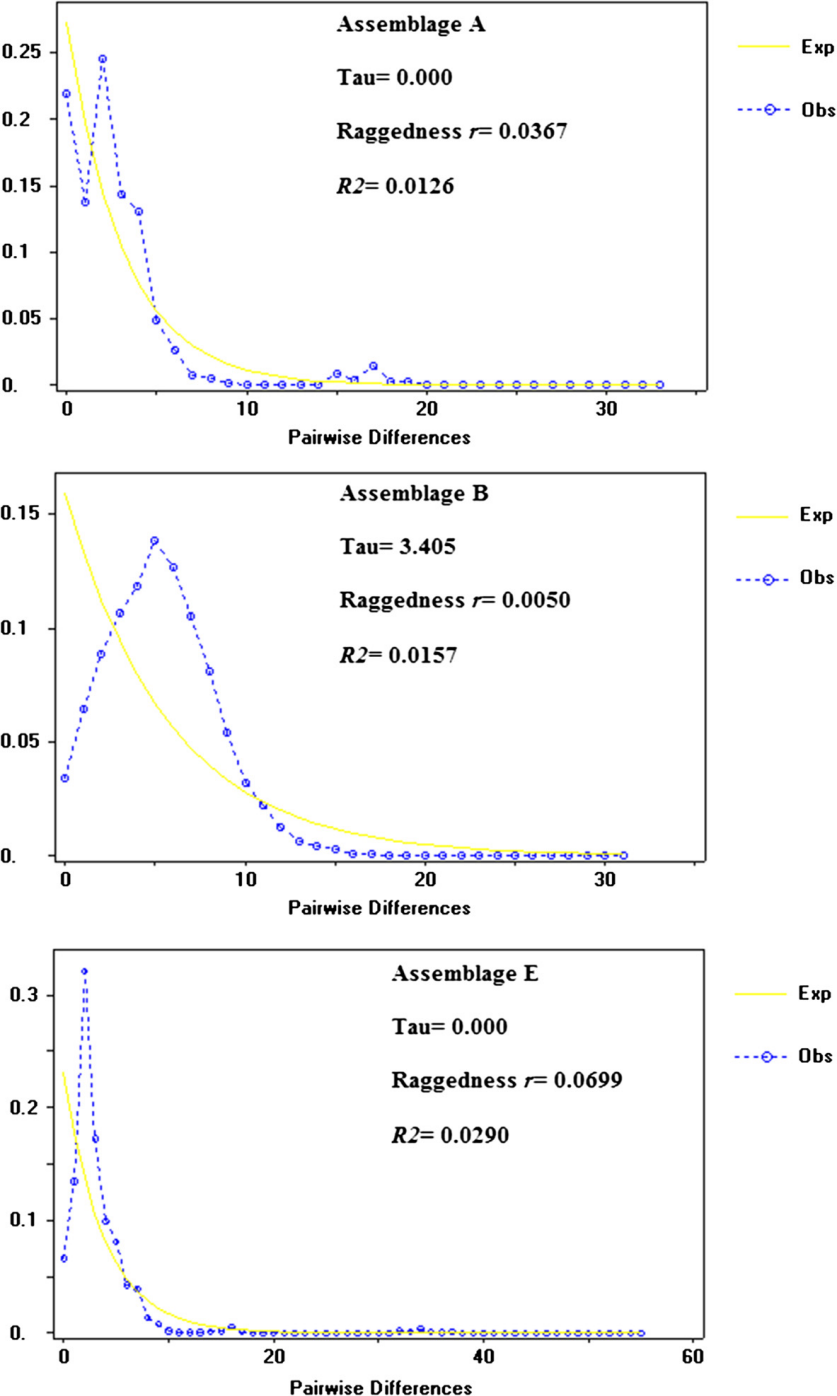
Observed and expected mismatch distribution for *Giardia duodenalis* based on *tpi* gene

Both of the statistical tests of neutrality, Tajima’s *D* and Fu and Li’s *D* produced consistent negative values for all assemblages and for all populations (Table 1). In most cases, the results of the test were statistically significant with the exception of Australian populations of all assemblages. This could be indicative of population size expansion and the hypothesis was supported by mismatch distribution test, which showed low values of the Raggedness index (P > 0.05) and R_2_ (P < 0.05) and unimodal mismatch distributions (Fig. 6).

### Genetic differentiation and gene flow

The level of genetic differentiation measured using the Wright’s test were determined for populations between each continent according to assemblage (Table 2). The gene flow or migration index (Nm) transformed from F_ST_ was also accessed and presented in Table 3.

**Table 3:** Genetic differentiation (FST) and gene flow (Nm)

Population 1	Population 2	Fst	Nm
Assemblage A			
Australia	America	0.01828*	13.43
Australia	Asia	0.35464*	0.45
Australia	Europe	0.13342*	1.62
Australia	Africa	0.17621	1.17
America	Asia	0.38293*	0.40
America	Europe	0.11833*	1.86
America	Africa	0.13786*	1.56
Asia	Europe	0.08993*	2.53
Asia	Africa	0.06020*	3.90
Europe	Africa	0.03860*	6.23
Total			1.50
Assemblage B			
Australia	America	0.09306	2.44
Australia	Asia	0.02193	11.15
Australia	Europe	0.05506	4.29
Australia	Africa	0.06653	3.51
America	Asia	0.05330*	4.44
America	Europe	0.01962	12.49
America	Africa	0.25445*	0.73
Asia	Europe	0.02529	9.63
Asia	Africa	0.11234	1.98
Europe	Africa	0.19843	1.01
Total			2.41
Assemblage E			
Australia	America	0.14462	1.48
Australia	Asia	0.10296*	2.18
Australia	Europe	0.02651	9.18
Australia	Africa	0.14812*	1.44
America	Asia	0.04029	5.96
America	Europe	0.25382	0.73
America	Africa	0.05736*	4.11
Asia	Europe	0.21494*	0.91
Asia	Africa	0.04215*	5.68
Europe	Africa	0.26526*	0.69
Total			1.48

Value with asterisks (*) indicate statically significant result (*p*-value < 0.05)

For assemblage A, huge differentiation levels were recorded between Asian population with Australian (F_ST_ = 0.35464, Nm = 0.45) and with American (F_ST_ = 0.38293, Nm = 0.40) populations. Genetic differentiation of negligible to moderate differentiation was estimated among the populations for assemblage B except that populations between America-Africa recorded level of differentiation of 0.25445 (Nm = 0.73). The level of genetic differentiation for assemblage E was above 0.15 and significant for populations between Europe-Africa (Nm = 0.69) and between Asia-Europe (Nm = 0.91) whereas the rest of the population pairs had negligible to moderate differentiation. The overall results showed moderate divergence among the populations with 25/30 of the population pairs had F_ST_ less than 0.25 and half of them were statistically significant. These results reflected the spread of the same population of *Giardia* isolates across the geographical regions. In general, gene flow levels were high (i.e. Nm > 1). The highest gene flow was observed in assemblage B (2.41) followed by assemblage A (1.50) and assemblage E (1.48).

## Discussion

In the present study, we investigate the population structure of *G. duodenalis* from Malaysian population and global sequence data based on *tpi* gene*.* To date, there is very little population genetic studies on this parasite, most of which gave emphasis in looking for evidence of sexual reproduction or recombination in *G. duodenalis* [20–22]. This study explored the genetic diversity of different assemblages distributed in different regions and measured the genetic structure in order to draw inferences regarding driving forces that control the evolutionary process of *G. duodenalis* and the potential factors that affect the dynamics and distribution of *Giardia* infection.

The level of genetic diversity was greater among the sequences of assemblage B compared to assemblage A for the Malaysian population. Comparison of Malaysian population with data from Thailand based on *bg* gene [23] showed that the nucleotide diversity was similar with Thailand i.e. 0.00235 vs. 0.00340 for assemblage A and 0.00433 vs. 0.00620 for assemblage B. Another study from Thailand based on *gdh* gene [24] also showed significant higher nucleotide divergence (K) in assemblage B than assemblage A. In the context of the overall worldwide comparison, assemblage B yielded the highest level of genetic diversity followed by assemblage E and assemblage A. Despite asexual reproduction having been generally regarded as the main reproduction mode for *Giardia*, the difference in genetic diversity are not clearly understood while some have suggested the different assemblages especially A and B to be considered as different species which also displayed some biological differences [25, 26]. With regards to epidemiological data that shown (allelic sequence heterozygosity) ASH reflected in the form of heterogeneous nucleotides particularly among the isolates of assemblage B, Andersson [27] proposes ameiotic crossing that has taken place in a more recent time either within nucleus or between nuclei before achieving homogenization is responsible for these observations. On the other hand, the low level of ASH among the isolates of assemblages A and E was regarded as a result of asexual reproduction that have been carried out long enough to purge out mutations and lose the allelic variation. A single clone that has reproduced and dispersed successfully in a population can potentially cause high resemblance between isolates.

In view of host specification, parasites with multiple hosts are less likely to experience local extinction and susceptible hosts can be easily found in new areas, thus should have comparatively higher levels of genetic variation [28]. Although both assemblages A and B infect humans and a wide range of animal hosts, there is marked difference with regard to host-specificity within sub-structuring of assemblages. Assemblage A comprises sub-assemblages AI, AII and AIII with host preferences in livestock, humans and wildlife respectively [29]. Whereas for assemblage B, the classification of sub-assemblages BIII and BIV was not reproducible and similar frequency was found in the distribution of the sub-genotype in human and animal hosts [29, 30]. While assemblage E infects only livestock, another possibility for the relative lower levels of genetic diversity in assemblage A and E could be due to the cycle of sub-assemblages A and assemblage E genotypes in limited hosts compared to assemblage B which has a broad host range and host-specificity in sub-assemblage that was not clearly exhibited.

The haplotype network analysis for assemblage A did not separate the isolates into networks according to geographical regions from where they were collected. It is worth noting that clustering of isolates into identified sub-assemblages (i.e. AI and AII) was obtained in the Malaysian population (Fig. 1), but such clustering was not seen in the global population despite the formation of three distinct networks (Fig. 3). All of the three networks contained isolates derived from humans and animals. In addition, it was found that humans were sharing the same haplotype with a wide range of animals such as cat, dog, cattle, sheep, alpaca, gull, grey seal and dolphin. A few studies [31, 32] had shown that phylogenetic network analysis can recognize species and sub-clustering within species. Regarding why known sub-clustering was only seen in Malaysian population of assemblage A but not in the global population as well as in the network analysis of assemblage B, Hart and Sunday [33] attributed the discordance between networks and taxa to several reasons such as inadequate sampling (e.g. sub-assemblage AIII), limited divergence, hybridization, cryptic speciation with undocumented phenotypic differences and incomplete lineage sorting. Similar to assemblage A, it was found that human and animal isolates of assemblage B were sharing the same haplotypes. On the other hand, animal isolates assemblage E did not share any haplotype with human. The sharing of the same haplotypes between animal and human may support the potential of zoonotic transmission of this parasite.

As a kind of parasite, *Giardia* is closely tied with its host as it is only in the intestine of the host where this flagellate can proliferate by binary fission. Moreover, the dispersal ability of this food and waterborne parasites is dependent on the host in which the cysts are spread and continue the lifecycle at other places. From the distribution pattern of haplotypes in the network analysis, corroborated with observation of moderate gene differentiation and high gene flow across continents, it was shown that *G. duodenalis* with similar genotype were well dispersed over the globe. In light of the rapid pace of globalization in sectors such as intercontinental travelling, migration, trading of livestock and agricultural activities, host dispersal can be a major determinant of parasite gene flow. Blouin et al. [34] in the population genetic study of nematodes had proposed host movement as the key role in gene flow of parasites which will in turn provide great opportunity in the dispersal of rare alleles e.g. drug resistant or virulent mutant alleles. Assemblage B which had high genetic diversity also had higher level gene flow than assemblages A and E. Pathogens with high gene flow generally have higher genetic diversity than those with low level of gene flow because high gene flow increases the size of the population and the geographical area where the genetic material is present [35]. The survival strategy of *Giardia* that is the formation of resistant cysts, enables the spread of the parasite genome over long distances and large areas. This genome dispersal is a kind of gene flow known as genotype flow which occurs in asexual reproduction pathogen (with little or no recombination) where entire genotypes can be transmitted from one population to another [35].

The negative sign shown in the tests of neutrality, Tajima’s *D* and Fu and Li’s *D* signifies an excess of low frequency of polymorphism compared to the expectations under neutral processes such as mutation, genetic drift and population size equilibrium [36]. In addition, the unimodal displayed in the mismatch distribution is an indication of populations that experience recent expansion. Populations that have been stable over time are expected to have a bimodal and multimodal mismatch distribution [37–39]. However, the influence of selection could be locus-dependent as test for neutrality from other studies suggested that the *bg* gene was possibly influenced by ongoing purifying selection [23] while the *gdh* gene was under neutral selection [24]. On the other hand, if *Giardia* undergoes reproduction asexually as it is generally assumed, the population would be made up of independently evolving lineages and mutations are expected to confine to the lineage in which they began [11], thereby the different rate of mutation with selection pressure might contribute to the excess polymorphism sites but low frequency of haplotype and limit its potential of evolution. Similar to the observation of high gene flow, recent expansion could have been promoted by human activities and host migrations.

As the population analyses were performed based on single gene, stronger inferences could be made if molecular data from multiple genes were included. In addition, it is well known that assemblages A and B can infect both humans and animals while assemblage E predominantly cause infection in livestock with a few rare cases reported in humans [29, 40]. Despite identical haplotypes being found shared by humans and animals, higher resolution of molecular data e.g. by combining sequences of different loci would be needed to provide sufficient evidence and understand the zoonotic potential of assemblages A and B. The complexity of life cycle, host specificity, modes of transmission and dispersal as well as the degree of recombination are the important history traits in shaping the population structure of microbial parasites [28, 41, 42].

## Conclusions

In general, high gene flow was observed in all of the assemblages and this could pose a threat in the transmission of the parasites especially the spread of the virulent alleles. The different genetic diversity observed in assemblages A, B and E could be due to their different evolutionary patterns especially between lineages of assemblages A and E with assemblage B. The departure from neutral expectations from the test results of Tajima’s *D* and Fu and Li’s *D* can be the consequence of population expansion after a bottleneck event or the presence of purifying selection. These findings provide fundamental evolutionary information of *G. duodenalis* and enhance our understanding of the dynamics and distribution of *Giardia* infection.

## References

[1] Ortega-Pierres G (2009). *Giardia* and *cryptosporidium*: from molecules to disease.

[2] Rivero FD, Muller M, Luján HD, Souza W (2010). Secretory events during *giardia* encystation. Structures and organelles in pathogenic protists. vol 17.

[3] Cavalier-Smith T (1987). Eukaryotes with no mitochondria. Nature.

[4] Koonin EV (2010). The origin and early evolution of eukaryotes in the light of phylogenomics. Genome Biol.

[5] Brinkmann H, Philippe H (2007). The diversity of eukaryotes and the root of the eukaryotic tree. Adv Exp Med Biol.

[6] Hjort K, Goldberg AV, Tsaousis AD, Hirt RP, Embley TM (2010). Diversity and reductive evolution of mitochondria among microbial eukaryotes. Philos Trans R Soc Lond B Biol Sci.

[7] Thompson RC, Monis PT (2004). Variation in *Giardia*: implications for taxonomy and epidemiology. Adv Parasitol.

[8] Alum A, Sbai B, Asaad H, Rubino JR, Khalid IM (2012). ECC-RT-PCR: a new method to determine the viability and infectivity of *Giardia* cysts. Int J Infect Dis.

[9] Feng YY, Xiao LH (2011). Zoonotic potential and molecular epidemiology of *Giardia* species and Giardiasis. Clin Microbiol Rev.

[10] Charles DC, Robert P, Michael SB. Molecular ecology of parasites: elucidating ecological and microevolutionary processes. Mol Ecol. 2005;14. 10.1111/j.1365-294X.2005.02587.x 15969711

[11] Andras JP, Ebert D (2013). A novel approach to parasite population genetics: experimental infection reveals geographic differentiation, recombination and host-mediated population structure in Pasteuria ramosa, a bacterial parasite of Daphnia. Mol Ecol.

[12] Choy SH, Al-Mekhlafi HM, Mahdy MA, Nasr NN, Sulaiman M, Lim YA (2014). Prevalence and associated risk factors of *Giardia* infection among indigenous communities in rural Malaysia. Sci Rep.

[13] Sulaiman IM, Fayer R, Bern C, Gilman RH, Trout JM, Schantz PM (2003). Triosephosphate isomerase gene characterization and potential zoonotic transmission of *Giardia duodenalis*. Emerg Infect Dis.

[14] Thompson JD, Higgins DG, Gibson TJ (1994). CLUSTAL W: improving the sensitivity of progressive multiple sequence alignment through sequence weighting, position-specific gap penalties and weight matrix choice. Nucleic Acids Res.

[15] Hall TA, editor. BioEdit: a user-friendly biological sequence alignment editor and analysis program for Windows 95/98/NT. Nucleic acids symposium series. California: Ibis Biosciences Carlsbad; 1999. http://www.mbio.ncsu.edu/BioEdit/bioedit.html

[16] Huson DH, Bryant D (2006). Application of phylogenetic networks in evolutionary studies. Mol Biol Evol.

[17] Librado P, Rozas J (2009). DnaSP v5: a software for comprehensive analysis of DNA polymorphism data. Bioinformatics.

[18] Wright S (1978). Evolution and the genetics of populations. Variability within and among natural populations.

[19] Govindajuru RD. Variation in gene flow levels among predominantly self-pollinated plants. J Evol Biol. 1989;2(3):173–181.

[20] Cooper MA, Adam RD, Worobey M, Sterling CR (2007). Population genetics provides evidence for recombination in *Giardia*. Curr Biol.

[21] Lasek-Nesselquist E, Welch DM, Thompson RC, Steuart RF, Sogin ML (2009). Genetic exchange within and between assemblages of *Giardia duodenalis*. J Eukaryot Microbiol.

[22] Takumi K, Swart A, Mank T, Lasek-Nesselquist E, Lebbad M, Caccio SM (2012). Population-based analyses of *Giardia duodenalis* is consistent with the clonal assemblage structure. Parasit Vectors.

[23] Kosuwin R, Putaporntip C, Pattanawong U, Jongwutiwes S (2010). Clonal diversity in *Giardia duodenalis* isolates from Thailand: evidences for intragenic recombination and purifying selection at the beta giardin locus. Gene.

[24] Siripattanapipong S, Leelayoova S, Mungthin M, Thompson RC, Boontanom P, Saksirisampant W (2011). Clonal diversity of the glutamate dehydrogenase gene in *Giardia duodenalis* from Thai isolates: evidence of genetic exchange or mixed infections?. BMC Microbiol.

[25] Franzen O, Jerlstrom-Hultqvist J, Castro E, Sherwood E, Ankarklev J, Reiner DS (2009). Draft genome sequencing of *Giardia intestinalis* assemblage B isolate GS: is human giardiasis caused by two different species?. PLoS Pathog.

[26] Xu F, Jerlstrom-Hultqvist J, Andersson JO (2012). Genome-wide analyses of recombination suggest that *Giardia intestinalis* assemblages represent different species. Mol Biol Evol.

[27] Andersson JO (2012). Double peaks reveal rare diplomonad sex. Trends Parasitol.

[28] Barrett LG, Thrall PH, Burdon JJ, Linde CC (2008). Life history determines genetic structure and evolutionary potential of host-parasite interactions. Trends Ecol Evol.

[29] Sprong H, Caccio SM, van der Giessen JW (2009). Identification of zoonotic genotypes of *Giardia duodenalis*. PLoS Negl Trop Dis.

[30] Wielinga CM, Thompson RC (2007). Comparative evaluation of *Giardia duodenalis* sequence data. Parasitology.

[31] Chen H, Strand M, Norenburg JL, Sun S, Kajihara H, Chernyshev AV (2010). Statistical parsimony networks and species assemblages in Cephalotrichid nemerteans (nemertea). PLoS One.

[32] Villalobos G, Orozco-Mosqueda G, Lopez-Perez M, Lopez-Escamilla E, Cordoba-Aguilar A, Rangel-Gamboa L (2014). Suitability of internal transcribed spacers (ITS) as markers for the population genetic structure of *Blastocystis* spp. Parasit Vectors.

[33] Hart MW, Sunday J (2007). Things fall apart: biological species form unconnected parsimony networks. Biol Lett.

[34] Blouin MS, Yowell CA, Courtney CH, Dame JB (1995). Host movement and the genetic structure of populations of parasitic nematodes. Genetics.

[35] Agrios GN (2005). Plant pathology.

[36] Teodorovic S, Braverman JM, Elmendorf HG (2007). Unusually low levels of genetic variation among *Giardia lamblia* isolates. Eukaryot Cell.

[37] Rogers AR, Harpending H (1992). Population growth makes waves in the distribution of pairwise genetic differences. Mol Biol Evol.

[38] Schneider S, Excoffier L (1999). Estimation of past demographic parameters from the distribution of pairwise differences when the mutation rates vary among sites: application to human mitochondrial DNA. Genetics.

[39] Slatkin M, Hudson RR (1991). Pairwise comparisons of mitochondrial DNA sequences in stable and exponentially growing populations. Genetics.

[40] Helmy YA, Klotz C, Wilking H, Krucken J, Nockler K, Von Samson-Himmelstjerna G (2014). Epidemiology of *Giardia duodenalis* infection in ruminant livestock and children in the Ismailia province of Egypt: insights by genetic characterization. Parasit Vectors.

[41] Criscione CD, Blouin MS (2004). Life cycles shape parasite evolution: comparative population genetics of salmon trematodes. Evolution.

[42] Tibayrenc M, Kjellberg F, Arnaud J, Oury B, Breniere SF, Darde ML (1991). Are eukaryotic microorganisms clonal or sexual? A population genetics vantage. Proc Natl Acad Sci U S A.

